# Altered GABA_A_ Receptor Expression in the Primary Somatosensory Cortex of a Mouse Model of Genetic Absence Epilepsy

**DOI:** 10.3390/ijms232415685

**Published:** 2022-12-10

**Authors:** Muhammad Hassan, Nadia K. Adotevi, Beulah Leitch

**Affiliations:** Department of Anatomy, School of Biomedical Sciences, and the Brain Health Research Centre, University of Otago, P.O. Box 913, Dunedin 9054, New Zealand

**Keywords:** GABA_A_ receptors, absence epilepsy, stargazer mouse, Western blotting, biochemical fractionation, immunogold-cytochemistry electron microscopy, cortico-thalamocortical network, primary somatosensory cortex

## Abstract

Absence seizures are hyperexcitations within the cortico-thalamocortical (CTC) network, however the underlying causative mechanisms at the cellular and molecular level are still being elucidated and appear to be multifactorial. Dysfunctional feed-forward inhibition (FFI) is implicated as one cause of absence seizures. Previously, we reported altered excitation onto parvalbumin-positive (PV^+^) interneurons in the CTC network of the stargazer mouse model of absence epilepsy. In addition, downstream changes in GABAergic neurotransmission have also been identified in this model. Our current study assessed whether dysfunctional FFI affects GABA_A_ receptor (GABA_A_R) subunit expression in the stargazer primary somatosensory cortex (SoCx). Global tissue expression of GABA_A_R subunits α1, α3, α4, α5, β2, β3, γ2 and δ were assessed using Western blotting (WB), while biochemically isolated subcellular fractions were assessed for the α and δ subunits. We found significant reductions in tissue and synaptic expression of GABA_A_R α1, 18% and 12.2%, respectively. However, immunogold-cytochemistry electron microscopy (ICC-EM), conducted to assess GABA_A_R α1 specifically at synapses between PV^+^ interneurons and their targets, showed no significant difference. These data demonstrate a loss of phasic GABA_A_R α1, indicating altered GABAergic inhibition which, coupled with dysfunctional FFI, could be one mechanism contributing to the generation or maintenance of absence seizures.

## 1. Introduction

Childhood absence epilepsy is a complex genetic neurological disorder characterized by generalized absence seizures, detected on electroencephalography (EEG) as hallmark 2.5–4 Hz spike-wave discharges (SWDs) [1,2]. It is a common pediatric syndrome accounting for nearly 10–17% of all childhood epilepsies [3]. SWDs are associated with hyperexcitations in the brain’s CTC network, which comprises reciprocal excitatory connections between the cortex and relay thalamus modulated by FFI PV^+^ inhibitory interneurons within the reticular thalamic nucleus (RTN) thalamus and cortex [1,4]. Pathological hyperexcitations can arise from disruptions in cellular and molecular activity within the CTC microcircuit network through multiple mechanisms. One such implicated mechanism involves impaired GABA_A_R expression caused by mutations in GABA_A_R subunit genes, such as in the α1 subunit gene [5,6] and R43Q mutation in the γ2 gene [7,8,9,10] observed in human patients. Significantly, the induction of these gene mutations in rodent models caused SWDs associated with altered GABA_A_R subunit expression and altered inhibition in the CTC network [11,12,13,14,15]. Although in some genetic absence seizure rodent models, such as the stargazer mouse, the primary genetic defect is not present in the GABA_A_Rs, these models often display secondary GABAergic changes thought to play a significant role in the generation or maintenance of SWDs [16].

The stargazer mouse presents with a primary genetic defect resulting in a deficit of stargazin [17,18], and we have previously demonstrated that this leads to a deficit of glutamatergic GluA4-containing α-amino-3-hydroxy-5-methyl-4-isoxazolepropionic acid (AMPA) receptors at excitatory synapses on PV^+^ feed-forward inhibitory interneurons of RTN [19], and primary SoCx [20,21,22]. Within the CTC network, activation of PV^+^ interneurons by cortical glutamatergic inputs at RTN manifests as FFI of thalamocortical relay neurons. Similarly, in the primary SoCx, activation of these interneurons by glutamatergic inputs from thalamocortical relay neurons leads to FFI of target principal neurons. Hence, seizure generation in the stargazer mouse model may be mediated by dysfunctional FFI, secondary to loss of AMPA receptors at PV^+^ interneurons, leading to disinhibition in the CTC network. This has been corroborated by our recent findings, where silencing PV^+^ interneurons in both the RTN and primary SoCx of mice, using Designer Receptors Exclusively Activated by Designer Drugs (DREADD) technology, induced absence-like SWDs [23], while activating them abolished pharmacologically induced SWDs [24].

The stargazin mutation is also associated with downstream changes in GABA_A_R subunit expression in multiple brain regions [25,26,27]. In the stargazer ventroposterior (VP) thalamic nucleus, we previously reported a specific increase in both tonic and phasic GABA_A_R subunits [28,29]. However, unlike the AMPA receptor loss, which is pre-seizure, the increase in GABA_A_R subunits expression in the thalamus occurs after the onset of seizures [30], and thus is unlikely to be causative of seizures, although a role in seizure maintenance should not be ruled out. GABA_A_Rs generate either phasic or tonic post-inhibitory currents depending on synaptic or extra-synaptic GABAergic activation, respectively [31]. Evidence suggests a variable change in phasic inhibition and increased tonic inhibition to be involved based on the animal model as well as the region of the CTC network observed [32]. Enhanced tonic inhibition in the VP thalamic relay nucleus due to GAT-1 malfunction has been identified as a potential source of SWDs [33,34]. However, this increase is detectable at varying periods in the different models and cannot be positively pinpointed as a cause or effect of the SWDs. The cortex has been suggested as the principal initiation site of the SWDs, with the initiation site focused on deep layers of the primary SoCx of rodent models [35,36,37,38]. Cortical initiation of seizures is also supported by EEG, magnetoencephalography (MEG), and functional magnetic resonance imaging evidence from human patients [1]. However, in the stargazer primary SoCx, GABA_A_R expression and function, which may have a role in the mechanisms of absence seizures, have not yet been investigated.

The rodent neocortex expresses GABA_A_R subunits α1–α5, β1–β3, γ2–γ3, and δ [39], with the subunit combination of synaptic α1β2γ2 by far the most abundantly expressed [40,41]. The principal mediator of potent and rapid inhibitory currents is the α1-containing phasic GABA_A_R [31,42,43], which is ubiquitously expressed in the cortex [44], with preferentially peri-somatic and peripheral dendritic expression on pyramidal neurons. These sites on pyramidal neurons are also convergence points for connectivity from fast-spiking PV^+^ interneurons [45]. To test our hypothesis that dysfunctional PV^+^ interneurons-mediated FFI could alter α1-containing synaptic GABA_A_R expression on target neurons to promote the initiation or maintenance of SWDs, we conducted a series of experiments, predominantly addressing changes in the ubiquitous GABA_A_R α1 subunit. Other relevant GABA_A_R subunits were also assessed for any concomitant changes. Collateral changes in subunits α3, β2, β3, and γ2 have been reported in other mouse models where the phasic GABA_A_R α1 subunit was compromised [12,13,46]. Since altered tonic inhibition has also been implicated in the generation of SWDs [34,47], stargazer primary SoCx was also probed for the α4, α5, and δ subunits, found predominantly in GABA_A_Rs mediating tonic inhibition [31]. Immunofluorescence confocal microscopy was conducted to confirm the cortical layer-specific distribution and PV^+^ cellular localization of GABA_A_R α1 in the primary SoCx of the homozygous epileptic (E, stg/stg) stargazers compared to control non-epileptic (NE; heterozygous [+/stg] and wild-type [+/+]) littermates. Semiquantitative WB analysis of whole primary SoCx was used to investigate tissue changes in GABA_A_R subunits α1, α3, α4, α5, β2, β3, γ2, and δ. Biochemical fractionation was then used to isolate synaptic and extra-synaptic fractions from the primary SoCx, which were subsequently probed for GABA_A_R subunits α1, α3, α4, α5, and δ. Finally, double-labelled ICC-EM was used to quantify relative levels of GABA_A_R α1 at synapses between PV^+^ interneurons and their targets in primary SoCx. 

## 2. Results

### 2.1. GABA_A_R α1 Is Expressed in all Primary SoCx Layers

**Figure 1:**
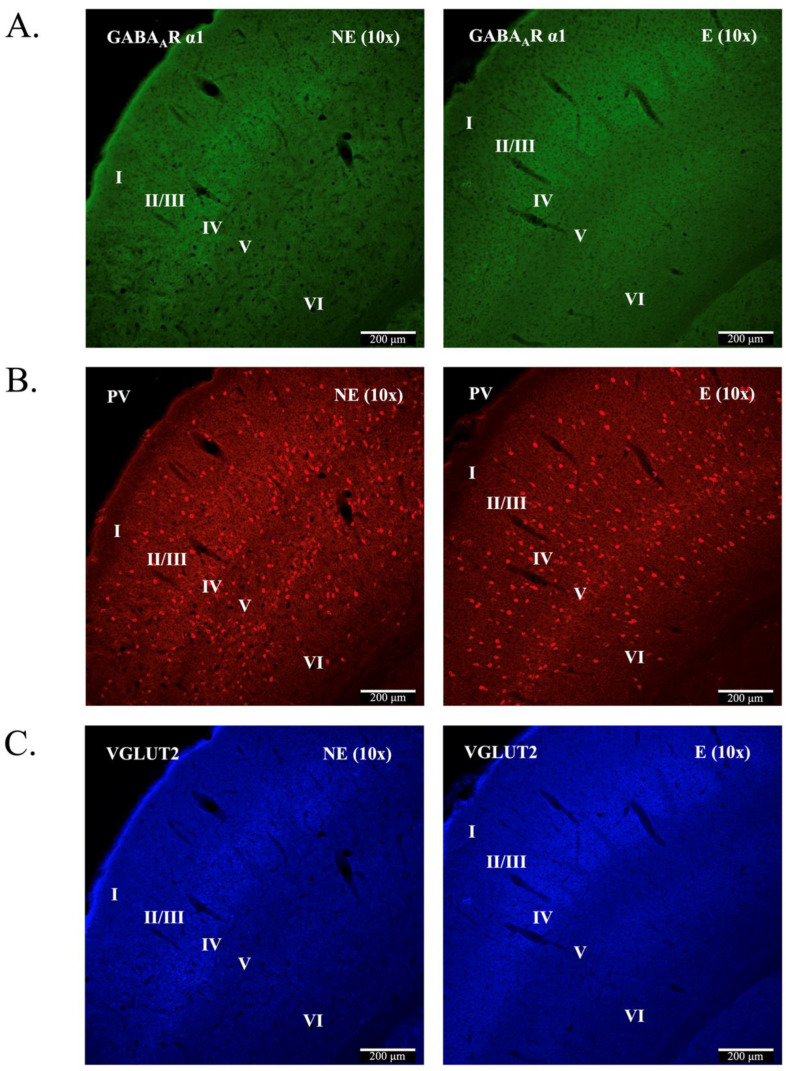
Representative confocal immunofluorescence images demonstrating expression of GABA_A_R α1 in the stargazer primary SoCx. 10× (Objective: 10× Plan) magnification from control (NE) littermate and epileptic (E) stargazer are shown here. (**A**) Pseudo-green channel shows diffuse labelling for GABA_A_R α1 subunit across the cortical layers with higher intensity in layer IV. (**B**) Pseudo-red channel shows labelling for PV^+^ neurons. The soma of PV^+^ neurons are present in all layers except layer I. (**C**) Pseudo-blue channel shows VGlut2 which assists in identification of the cortical layers. Intense labelling can be seen, predominantly, in layer IV due to the presence of thalamocortical excitatory nerve terminals.

**Figure 2:**
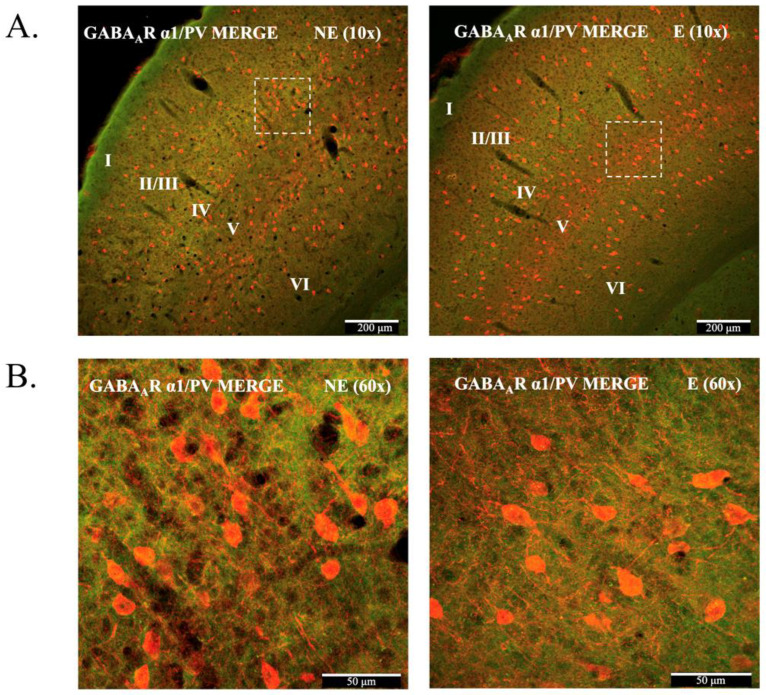
Merged confocal immunofluorescence images demonstrating the co-labelling for GABA_A_R α1 and PV in the primary SoCx of control (NE) littermate and epileptic (E) stargazer. (**A**) 10× magnification of GABA_A_R α1/PV merged image shows co-localization of GABA_A_R α1 with PV^+^ somas and processes. **(Β)** 60× (Objective: 60× PlanApo oil) magnification of GABA_A_R α1/PV merged image clearly shows GABA_A_R α1 present throughout the cortex as well on PV somas.

Confocal immunofluorescence (Figure 1A–C) confirmed the presence of GABA_A_R α1 in all layers of the primary SoCx in the control littermates and stargazers (Figure 1A). GABA_A_R α1 immunofluorescence displayed nearly uniform distribution across cortical layers, with relatively higher intensity in layer IV, identified by labelling with VGlut2 (Figure 1C), which intensely labels excitatory thalamocortical terminals projecting to cortical layer IV [48,49,50,51]. This distribution pattern of GABA_A_R α1 across cortical layers has been reported previously [39,52]. PV^+^ somas were seen in all layers except layer 1 (Figure 1B), as previously reported [53,54,55]. Higher numbers were apparent in layers IV and V, which concurs with our previously published report [21]. Figure 2 shows merged images for PV and GABA_A_R α1, demonstrating co-labelling of GABA_A_R α1 and PV^+^ in PV^+^ somas as well as the surrounding neuropil, which agrees with published reports [56]. Both GABA_A_R α1 pre-adsorption and omission controls showed virtually no fluorescence (Supplementary Figure S1), which confirmed the specificity of the GABA_A_R α1 antibody. 

### 2.2. Reduced GABA_A_R α1 Levels in the Stargazer Primary SoCx with no Change Seen in Other Subunits

**Figure 3:**
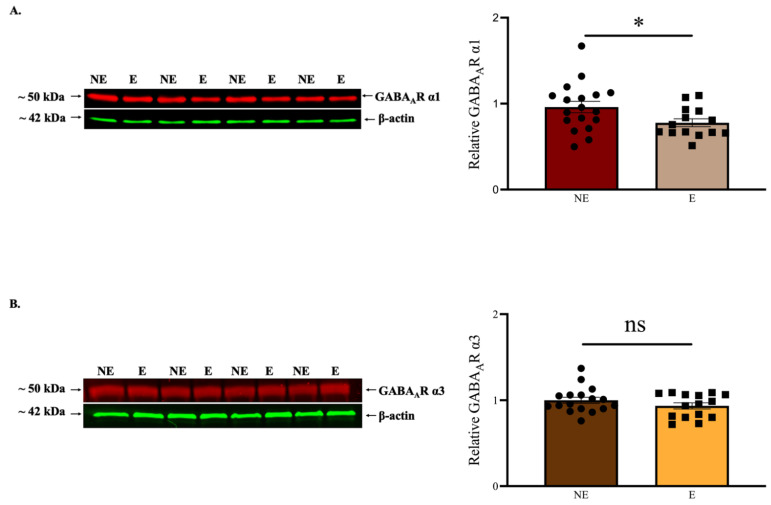
WB analysis for GABA_A_R α1 and α3 subunits in the primary SoCx. Data compare expression between control (NE) littermates and epileptic (E) stargazers. (**A**) Representative blots display GABA_A_R α1 in the primary SoCx with β-actin as the loading control. Bar graphs represent the relative expression levels of GABA_A_R α1 (mean ± SEM). WB analysis revealed a statistically significant reduction in whole-tissue expression levels of GABA_A_R α1 in the primary SoCx of the stargazers (NE: 0.960 ± 0.066 [*n* = 18], E: 0.78 ± 0.046 [*n* = 14], *p* = 0.041); (**B**) Representative blots display GABA_A_R α3 in the primary SoCx with β-actin as the loading control. Bar graphs represent the relative expression levels of GABA_A_R α3. There was no statistically significant change in whole-tissue expression levels of GABA_A_R α3 in the primary SoCx of the stargazers (NE: 1.000 ± 0.036 [*n* = 17], E: 0.935 ± 0.049 [*n* = 15], *p* = 0.519). The significance threshold was set at 0.05 with * indicating *p* < 0.05. ‘ns’ indicates no significant change found from analyses.

Expression levels of GABA_A_R α1 subunit were compared in the stargazer primary SoCx and their control NE littermates using semi-quantitative WB analysis. WB scans revealed bands at the expected molecular weights of ~50 kDa for GABA_A_R α1 and ~42 kDa for β-actin as recognized from the protein ladder (Figure 3A). The band intensities for GABA_A_R α1 were normalized against β-actin intensities. The results indicated a significant reduction of 18% in the tissue expression levels of GABA_A_R α1 (NE: 0.960 ± 0.066 [*n* = 18], E: 0.78 ± 0.046 [*n* = 14], *p* = 0.041) in the primary SoCx of the stargazers compared to their NE littermates (Figure 3A). GABA_A_R α3 levels, which have been reported to show a compensatory increase in response to loss of GABA_A_R α1 [12,46], were not significantly different (NE: 1.000 ± 0.036 [*n* = 17], E: 0.935 ± 0.049 [*n* = 15], *p* = 0.519) in stargazers compared to control littermates (Figure 3B).

**Figure 4:**
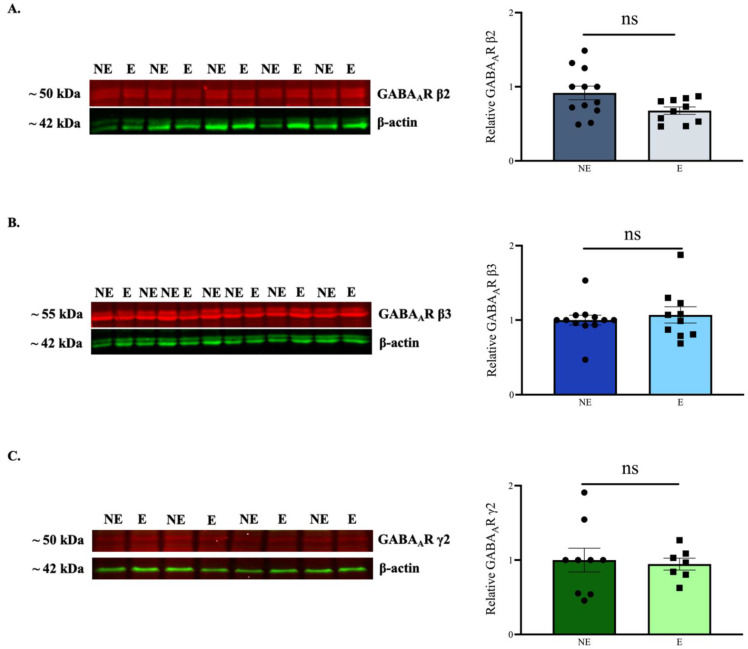
WB analysis for GABA_A_R β2, β3, and γ2 subunits in the primary SoCx. The data compare expression between control (NE) littermates and epileptic (E) stargazers. (**A**–**C**) Representative blots display tissue expression of GABA_A_R β2, β3, and γ2 subunits in the primary SoCx with β-actin as the loading control. Bar graphs represent the relative expression levels of GABA_A_R β2, β3, and γ2 subunits, respectively. Bar graphs show a lack of statistically significant change in whole-tissue primary SoCx expression levels for β2 (NE: 0.917 ± 0.091 [*n* = 12], E: 0.678 ± 0.050 [*n* = 10]; *p* = 0.093), β3 (NE: 1.000 ± 0.067 [*n* = 12], E: 1.070 ± 0.109 [*n* = 10]; *p* = 0.859), and γ2 (NE: 1.000 ± 0.159 [*n* = 9], E: 0.946 ± 0.079 [*n* = 7]; *p* = 0.900). The significance threshold was set at 0.05. ‘ns’ indicates no significant change found from analyses.

GABA_A_R β2, β3 and γ2 subunits are required for the arrangement of a functional pentameric α1-containing phasic GABA_A_R [57,58]. In addition, these subunits are also functionally expressed in tonic GABA_A_Rs combinations. Reduced expression of GABA_A_R α1 in knock-out models of absence seizures is accompanied by concurrent changes in these subunits as well [12,46]. However, in the current study semi-quantitative WB analysis of whole-tissue lysate did not reveal any significant changes in the levels of β2 (NE: 0.917 ± 0.091 [*n* = 12], E: 0.678 ± 0.050 [*n* = 10]; *p* = 0.093), β3 (NE: 1.000 ± 0.067 [*n* = 12], E: 1.070 ± 0.109 [*n* = 10]; *p* = 0.859) and γ2 (NE: 1.000 ± 0.159 [*n* = 9], E: 0.946 ± 0.079 [*n* = 7]; *p* = 0.900) subunits in the stargazers compared to their NE littermates (Figure 4A–C).

**Figure 5:**
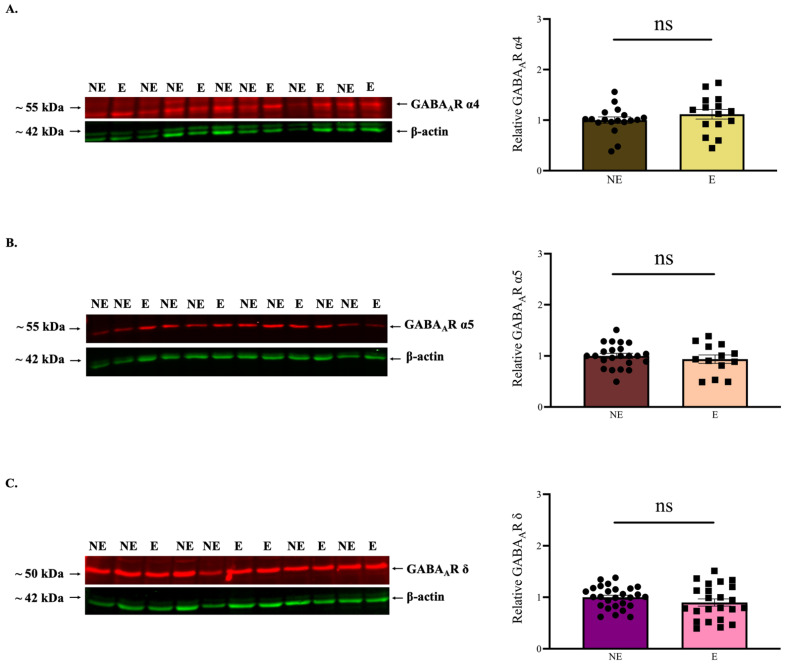
WB analysis for the principal tonic GABA_A_R α4, α5, and δ subunits. Analysis compares the primary SoCx of control (NE) littermates and the epileptic (E) stargazers. (**A**–**C**) Representative blots display tissue expression of GABA_A_R α4, α5, and δ subunits in the primary SoCx with β-actin as the loading control. Bar graphs represent the relative expression levels of GABA_A_R α4, α5 and δ subunits, respectively. Bar graphs reveal a lack of statistically significant change in whole-tissue primary SoCx expression levels for GABA_A_R α4 (NE: 1.000 ± 0.063 [*n* = 18], E: 1.117 ± 0.095 [*n* = 15]; *p* = 0.325), α5 (NE: 1.000 ± 0.048 [*n* = 23], E: 0.937 ± 0.083 [*n* = 13]; *p* = 0.672), and δ (NE: 1.000 ± 0.041 [*n* = 27], E: 0.899 ± 0.069 [*n* = 23]; *p* = 0.215). The significance threshold was set at 0.05. ‘ns’ indicates no significant change found from analyses.

A recent report described the suppression of SWDs in the stargazers after an effective knock-out of GABA_A_R δ subunit [47], which is expressed in GABA_A_Rs that mediate tonic inhibition [31]. Abnormal tonic inhibition in the stargazer VP thalamic nucleus has previously been reported [34]. Moreover, we also reported an increase in tonic GABA_A_R subunits α4 and δ levels in the VP thalamic nucleus, but only after the onset of seizures in the stargazers [28,30]. To assess whether any changes in tonic GABA_A_R subunits occur in the adult stargazer primary SoCx, we conducted further semiquantitative WB analyses to investigate levels of GABA_A_R α4, α5, and δ subunits. No significant changes in α4 (NE: 1.000 ± 0.063 [*n* = 18], E: 1.117 ± 0.095 [*n* = 15]; *p* = 0.325), α5 (NE: 1.000 ± 0.048 [*n* = 23], E: 0.937 ± 0.083 [*n* = 13]; *p* = 0.672), or δ (NE: 1.000 ± 0.041 [*n* = 27], E: 0.899 ± 0.069 [*n* = 23]; *p* = 0.215) subunits were seen in the stargazer primary SoCx compared to the control littermates (Figure 5A–C). 

### 2.3. Verification of the Biochemical Fractionation Technique

**Figure 6:**
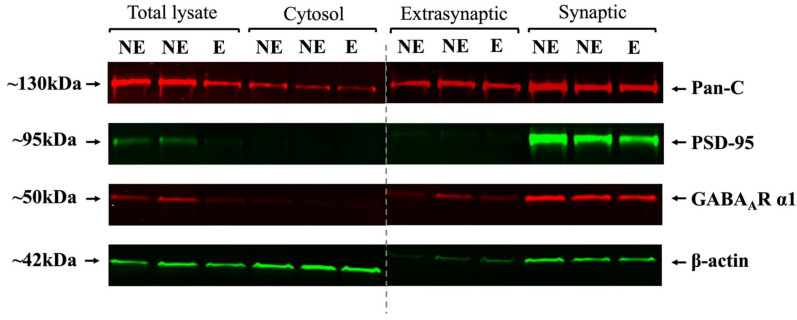
Pilot WB run for biochemically isolated primary SoCx subcellular fractions (total lysate, cytosol, extra-synaptic and synaptic). This run confirmed the successful isolation of the synaptic fractions given the intense labelling for both PSD95 and GABA_A_R α1. Extra-synaptic fraction, on the other hand, showed no bands for PSD95, but low intensity bands were seen for GABA_A_R α1 and β-actin. PanC showed good expression in all subcellular fractions.

A pilot study was first conducted to validate the purity of the biochemically fractionated subcellular components. Figure 6 shows a representative Western blot of the four subcellular fractions with immunofluorescence bands detected at the specified molecular weights of 130 kDa for PanC, 95 kDa for PSD95, 50 kDa for GABA_A_R α1, and 42 kDa for β-actin. PanC was detected in all fractions as expected [59], whereas PSD95, a synaptic scaffold protein [20,60,61], was only present in the synaptic component and not in the extra-synaptic fraction, thus confirming the purity of the synaptic fraction. Signal values for the subunits were normalized against Pan-C, as this protein is not associated with the primary mutation in the stargazers and has been shown to remain unchanged across subcellular fractions compared to their control littermates [20,62,63]. β-actin showed variable enrichment in the four synaptic components and was not used as a normalization protein in this instance due to its inconsistent expression and association with membranous components [20,62,64]. As expected, GABA_A_R α1 displayed the highest intensity in the synaptic fraction where it is known to be predominantly expressed for fast phasic GABAergic neurotransmission. 

### 2.4. Synaptic GABA_A_R α1 Is Significantly Reduced in Stargazer Primary SoCx with No Significant Subcellular Change in Other GABA_A_R Subunits

**Figure 7:**
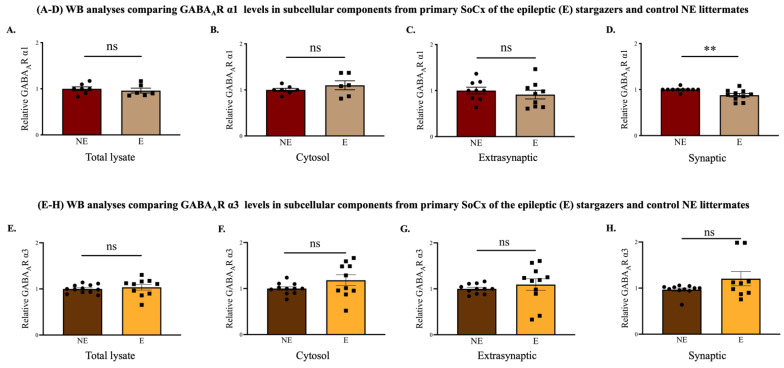
WB analyses of biochemically isolated fractions from the primary SoCx for GABA_A_R α1 and α3 subunits. Isolated subcellular fractions were compared between control (NE) littermates and epileptic (E) stargazers. (**A**–**D**) Biochemical fractionation analysis revealed a 12.2% reduction in the synaptic expression of phasic GABA_A_R α1 in the primary SoCx of the stargazers compared to their control littermates (NE: 1.000 ± 0.146 [*n* = 10], E: 0.878 ± 0.037 [*n* = 10]; *p* = 0.002); no significant difference was revealed from the other subcellular components: total lysate (NE: 1.000 ± 0.043 [*n* = 7], E: 0.961 ± 0.052 [*n* = 6]; *p* = 0.531), cytosol (NE: 1.000 ± 0.034 [*n* = 7], E: 1.100 ± 0.098 [*n* = 6]; *p* = 0.443), and extra-synaptic (NE: 1.000 ± 0.074 [*n* = 9], E: 0.912 ± 0.094 [*n* = 9]; *p* = 0.385). (**E**–**H**) Biochemical fractionation analysis revealed no significant change in GABA_A_R α3 in all subcellular components from the primary SoCx of stargazers compared to their control littermates: Total lysate (NE: 1.000 ± 0.026 [*n* = 12], E: 1.035 ± 0.060 [*n* = 10]; *p* = 0.381), cytosol (NE: 1.000 ± 0.037 [*n* = 11], E: 1.181 ± 0.118 [*n* = 10]; *p* = 0.349), extra-synaptic (NE: 1.000 ± 0.031 [*n* = 11], E: 1.093 ± 0.125 [*n* = 11]; *p* = 0.133), and synaptic (NE: 0.967 ± 0.034 [*n* = 11], E: 1.205 ± 0.153 [*n* = 9], *p* = 0.359). The significance threshold was set at 0.05 with ** indicating *p* < 0.01. ‘ns’ indicates no significant change found from analyses.

**Figure 8:**
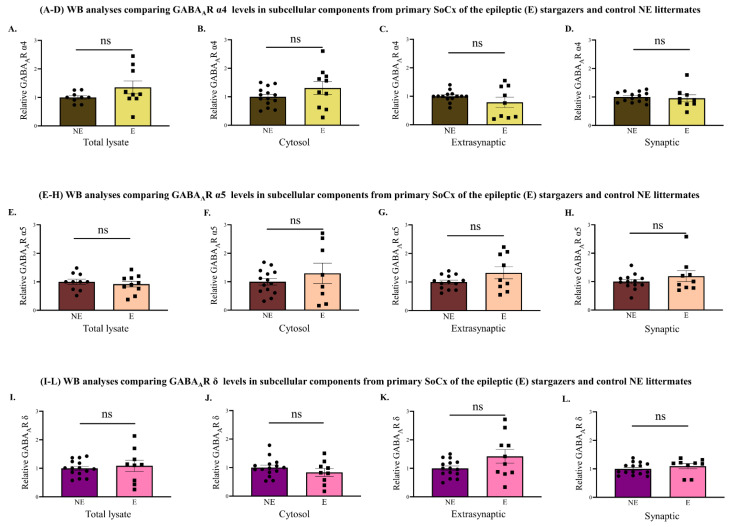
WB analyses of biochemically isolated fractions from primary SoCx for GABA_A_R α4, α5, and δ subunits. Subcellular fractions were isolated from the control (NE) littermates and epileptic epileptic (E) stargazers. (**A**–**D**) Biochemical fractionation analysis revealed no significant change in GABA_A_R α4 in all subcellular components from the primary SoCx of stargazers compared to their NE littermates: total lysate (NE: 1.000 ± 0.063 [*n* = 9], E: 1.353 ± 0.227 [*n* = 9]; *p* = 0.257), cytosol (NE: 1.000 ± 0.088 [*n* = 14], E: 1.308 ± 0.223 [*n* = 10]; *p* = 0.172), extra-synaptic (NE: 1.000 ± 0.051 [*n* = 14], E: 0.789 ± 0.183 [*n* = 9]; *p* = 0.516), and synaptic (NE: 1.000 ± 0.047 [*n* = 14], E: 0.955 ± 0.122 [*n* = 9]; *p* = 0.369). (**E**–**H**) GABA_A_R α5 analysis revealed no significant change in all subcellular components from the primary SoCx of stargazers compared to their control littermates: total lysate (NE: 1.000 ± 0.094 [*n* = 10], E: 0.919 ± 0.102 [*n* = 10]; *p* = 0.566), cytosol (NE: 1.000 ± 0.113 [*n* = 14], E: 1.295 ± 0.362 [*n* = 8]; *p* = 0.920), extra-synaptic (NE: 1.000 ± 0.068 [*n* = 13], E: 1.324 ± 0.214 [*n* = 9]; *p* = 0.431) and, synaptic (NE: 1.000 ± 0.075 [*n* = 13], E: 1.190 ± 0.195 [*n* = 9]; *p* = 0.744). (**I**–**L**) No statistically significant change was also seen for GABA_A_R δ subunit in all subcellular components from the primary SoCx of stargazers compared to their control littermates: total lysate (NE: 1.000 ± 0.071 [*n* = 15], E: 1.088 ± 0.201 [*n* = 9]; *p* = 0.815), cytosol (NE: 1.000 ± 0.083 [*n* = 15], E: 0.831 ± 0.138 [*n* = 9]; *p* = 0.379), extra-synaptic (NE: 1.000 ± 0.078 [*n* = 15], E: 1.419 ± 0.241 [*n* = 10]; *p* = 0.191), and synaptic (NE: 1.000 ± 0.053 [*n* = 15], E: 1.092 ± 0.094 [*n* = 9]; *p* = 0.263). The significance threshold was set at 0.05. ‘ns’ indicates no significant change found from analyses.

Since the stargazer primary SoCx showed a tissue reduction of GABA_A_R α1, it was necessary to determine whether such loss occurred in the synapses of the stargazer primary SoCx. For this purpose, biochemical fractionation technique was used to obtain subcellular fractions from primary SoCx homogenates followed by probing for GABA_A_R α1 using WB analysis. WB analysis of the synaptic fraction revealed a statistically significant reduction of 12.2 percent in GABA_A_R α1 subunit expression in stargazers compared to their NE littermates (NE: 1.000 ± 0.146 [*n* = 10], E: 0.878 ± 0.037 [*n* = 10]; *p* = 0.002; Figure 7D). No difference was detected in GABA_A_R in the other three subcellular fractions (Figure 7A–C). Biochemically isolated subcellular fractions were also probed for GABA_A_R α3, α4, α5, and δ. This was to reveal any altered levels that may have been masked upon WB analysis of global tissue expression. There was no significant change in the α3 subunit in any subcellular fraction from stargazer primary SoCx compared to their control littermates (Figure 7E–H). Our results also revealed no significant change in either α4 (Figure 8A–D), α5 (Figure 8E–H), or δ (Figure 8I–L) subunit levels in all the sub-cellular fractions (total lysate, cytosol, synaptic, and extra-synaptic). 

### 2.5. PV^+^ Feed-Forward Inhibitory Interneurons Show No Overall Change in Synaptic GABA_A_R α1

**Figure 9:**
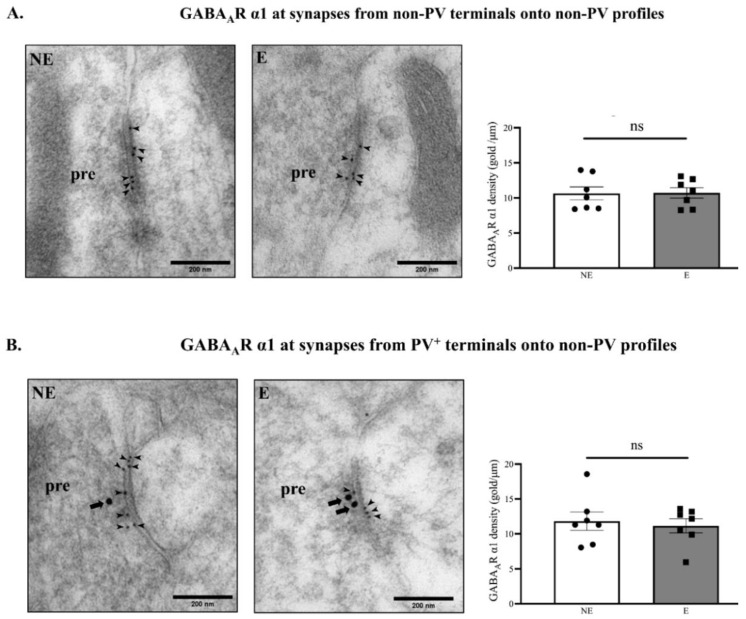
ICC-EM analysis for GABA_A_R α1 at PV^+^ and non-PV inhibitory synapses in the primary SoCx compared and analyzed between control (NE) littermates and epileptic (E) stargazers. From top to bottom: Representative EM micrographs demonstrate PV labelling in the presynaptic terminals (pre), which appear darker stained, in stargazer and NE littermate primary SoCx. Arrow represents 20 nm immunogold particle labelling PV while arrowhead represents 10 nm immunogold labelling GABA_A_R α1. (**A**) For analysis for GABA_A_R α1 at inhibitory synapses from non-PV terminals onto non-PV profiles, as indicated by lack of 20 nm gold particles, 490 synapses in stargazers and 490 in control littermates primary SoCx were analyzed from seven pairs distributed equally. No significant difference was seen in the seven pairs (NE: 10.65 ± 0.918 [*n* = 7], E: 10.72 ± 0.751 [*n* = 7]; *p* = 0.804). (**B**) Analysis for GABA_A_R α1 at inhibitory synapses with PV (20 nm gold) in the presynaptic terminals revealed no significant difference between the stargazers (63 synapses analyzed) and control littermates (63 synapses analyzed) (NE: 11.82 ± 1.325 [*n* = 7], E: 11.15 ± 1.007 [*n* = 7], *p* > 0.999). The significance threshold was set at 0.05. ‘ns’ indicates no significant change found from analyses.

Since biochemical fractionation revealed that GABA_A_R α1 was significantly reduced in synaptic fractions from stargazers, the next step was to determine if the subunit was reduced specifically at synapses between PV^+^ feed-forward interneurons and their targets. PV^+^ neurons synapse onto the soma, proximal dendrites, and axonal initial regions of the pyramidal cells [65], which are their primary targets in the SoCx. However, they also show a high degree of reciprocal connectivity with each other and other inhibitory interneurons [66]. Double-labelled ICC-EM of primary SoCx sections with 10 nm immunogold for GABA_A_R α1 and 20 nm gold for PV were used to detect GABA_A_R α1 at inhibitory synapses between PV^+^ interneurons and their targets. The majority of the inhibitory synapses imaged did not have 20 nm gold particle labelling in both pre- and post-synaptic profiles. At the inhibitory synapses between these non-PV labelled neurons in the stargazer primary SoCx, there was no significant difference in the density of 10 nm gold particles label for GABA_A_R α1 (Figure 9A; NE: 10.65 ± 0.918 [*n* = 7], E: 10.72 ± 0.751 [*n* = 7]; *p* = 0.804). Gold particles/μm at inhibitory synapses, between PV^+^ feed-forward inhibitory terminals and their primary targets, were assessed by identifying GABA_A_R α1 10 nm gold-labelled synapses where PV 20 nm immunogold was restricted to presynaptic terminals. Inhibitory presynaptic terminals were identified by the presence of pleiomorphic vesicles observed as a denser and darker stained profile [67]. The combined data from all seven pairs revealed no significant difference in GABA_A_R α1 levels at output synapses from PV^+^ feed-forward inhibitory neurons onto their non-PV targets (Figure 9B; NE: 11.82 ± 1.325, E: 11.15 ± 1.007, *p* > 0.999). 

## 3. Discussion

In this study, findings revealed for the first time a significant reduction in synaptic GABA_A_R α1 in the primary SoCx of the stargazer mouse model of absence epilepsy. Since synaptic GABA_A_R α1 subunit is crucial for rapid inhibitory post-synaptic potentials [31,42,43], this suggests a potential reduction in overall phasic inhibition in the stargazer primary SoCx. Such a reduction would suggest an increase in the overall cortical hyperexcitability, which may contribute to the generation or maintenance of seizures. Our results also suggest a potential restructuring in the cortical GABAergic organization as our EM results revealed no significant change in GABA_A_R α1 levels at synapses between PV^+^ interneurons and their targets. No concurrent changes were found in other GABA_A_R subunits investigated in this study, which included α3, α4, α5, β2, β3, γ2, and δ. It is possible that efficient reorganization of the subunits in GABA_A_R pentamer masks any changes and thus were not detectable with the techniques utilized in this study.

### 3.1. Altered Synaptic GABA_A_R α1 Expression in the Stargazer Primary SoCx

In the current study, we report a statistically significant but small reduction only in the synaptic expression of GABA_A_R α1 subunit in the primary SoCx of the stargazer mouse model. We found no concurrent changes in α1 expression at either extra-synaptic site where it is also found [68], or intracellularly, where synthesis and protein assembly occur. This differs from findings in the heterozygous GABA_A_R α1 knock-out mouse model of absence epilepsy where a concurrent reduction in tissue and surface expression of the subunit has been reported [12]. Furthermore, in the GABA_A_R α1 knock-out mouse model of absence epilepsy [12,46], a loss of the α1-subunit is associated with altered expression of other GABA_A_R subunits co-expressed with α1, suggesting a rearrangement of the pentameric structure inevitably resulting in altered inhibitory dynamics. In such mice, concurrent increases in cortical levels of α2 and α3, reductions in cortical levels of GABA_A_R β2, β3, and γ2, and cortical layer-specific reduction in peak inhibitory postsynaptic currents have been reported [12,46]. Similar concurrent changes in GABA_A_R subunits have also been reported in the Wistar Albino Glaxo from Rijswijk rat (WAG/Rij) which shows significant reduction in cortical GABA_A_R α1, β3, and γ2 [69], perhaps correlated to the significant reduction in cortical inhibition reported in these rats [70,71]. In the stargazer primary SoCx, however, similar changes were not seen in the GABA_A_R subunits (α3, β2, β3, and γ2) investigated that show co-expression with synaptic GABA_A_R α1. A reason for this could that the marginal reduction in α1 induces only modest compensatory changes in the other synaptic GABA_A_R subunits, such that these changes are not detectable as significantly altered expressions. 

Synaptic α1-containing GABA_A_Rs are the principal mediators of fast synaptic inhibition [31,42,43], comprising nearly 40–60% of cortical GABA_A_Rs with preferential peri-somatic and peripheral dendritic expression at PV^+^ inputs onto principal pyramidal neurons and other PV^+^ interneurons [42,72]. GABA_A_R α1 subunit loss on principal pyramidal neurons would potentially increase their excitability. Conversely, such a loss on PV^+^ somas and dendrites themselves would potentially cause increased inhibition of target pyramidal neurons due to the disinhibitory effect in PV^+^ interneurons. Reports suggest that downregulation of α1-containing GABA_A_Rs and GABAergic inputs, in response to reduced excitatory input on inhibitory interneurons, preferentially occurs in pyramidal neurons [73,74]. In the stargazer, a loss in GABA_A_R α1 in pyramidal neurons could be attributed to a downstream effect of dysfunctional PV^+^ inhibitory interneurons or even increased internalization of GABA_A_Rs in response to enhanced neuronal excitability [75]. 

### 3.2. GABA_A_R α1 Is Not Altered at Synapses from PV^+^ Interneurons Onto Targets

Although the current study discovered a loss of synaptic GABA_A_R α1 in the stargazer primary SoCx when analyzed in the whole tissue protein homogenate, results from EM revealed no change in α1 density within each inhibitory synapse from PV^+^ terminals onto non-PV targets. It is highly possible that altered α1 subunit density within inhibitory synapses is cortical layer-specific, which was not investigated in this study. Cortical layer-specific changes in inhibition have been reported in other rodent models of absence seizures [11,76,77]. A second possibility is a change in the morphology of GABAergic neurons as a result of loss of excitatory input, which potentially alters the number of synapses but not the density of α1 subunit within each synapse. Such a reduction has been reported in the stargazer cerebellum as well [78]. Indeed, altered strength of excitatory inputs can bring adaptive changes in inhibitory GABAergic networks [79], which could potentially augment the loss of appropriate inhibitory inputs onto target neurons, leading to other downstream changes because of increased excitability. Developmentally, in mice, fast-spiking activity of PV^+^ interneurons engage by day 18, which interestingly in stargazers coincides with the onset of SWDs [17]. It is likely that in the stargazers, dysfunctional PV^+^-mediated FFI triggers mechanisms that translate into reduced overall GABA_A_R α1 expression. It should be emphasized that although synaptic GABA_A_R α1 is largely expressed around PV^+^ interneurons and is likely reduced around these interneurons, given the stargazer’s dysfunctional PV^+^-mediated FFI, the possibility of differential effects between other populations of cortical neurons cannot be excluded.

### 3.3. Impact of Altered Cortical Inhibition

Recently members of our lab demonstrated that independently silencing PV^+^ interneurons in both the cortex and RTN using DREADD technology in freely moving mice produced absence-like SWDs [23,80]. This recaptures the dysfunctional FFI of the stargazer mouse where PV^+^ interneurons of the CTC network are affected by loss of AMPA receptors [20,21,22]. However, unlike acutely induced change in the DREADD mice, the stargazers present with a chronic loss of excitation (via loss of AMPA receptors activity) at PV^+^ interneurons concurrently in the primary SoCx and the RTN. In the RTN, increased activity of NMDA receptors has been reported in stargazers [81], albeit in the absence of altered NMDA receptors expression [63], perhaps as a compensatory glutamatergic activation of NMDA receptors in the absence of AMPA receptors [82]. Likewise, the increase in GABA_A_Rs subunits expression in the thalamic relay neurons post-seizure onset [28,29,30] may also be a compensatory consequence of reduced RTN-mediated inhibition, which preferentially targets VP thalamic nucleus [83]. In this context, recently it was reported that in the freely moving Genetic Absence Epilepsy Rat from Strasbourg (GAERS), the large majority of thalamocortical relay neurons remain silent during SWDs with few displaying increased phasic and tonic inhibition as a result of burst activity from the RTN, which is activated by top-down cortical excitations [37]. Indeed, recent computational modelling studies further demonstrate the suppressive role of intra-thalamic phasic and tonic inhibition during SWDs [84]. 

As for the SoCx, reports from different animal models suggest both enhanced inhibition as well as reduced inhibitory activity [16,32]. Layer-specific reduced inhibition has been reported in various animal models including: the WAG/Rij, which displays reduced expression of PV^+^ interneurons in the primary SoCx [76]; a mouse model of a human genetic absence epilepsy syndrome [12], which shows reduced inhibitory postsynaptic currents in layer VI of sensory cortex; and the knock-in γ2R43Q mouse model with a point mutation in GABA_A_R γ2, which presents with reduced inhibitory currents in pyramidal neurons [11]. In the GAERS, however, reports suggest an increase in cortical inhibition as a result of increased cortical GABA_B_ receptor expression, which contributes to tonic inhibition [34,85]. GAERS also have layer-specific increases in PV^+^ (layer V) and SOM^+^ (Layer IV) inhibitory interneurons [76,77], and increased expression of stargazin and AMPA receptors [86,87]. It should be noted that increased inhibition in the cortex could paradoxically lead to increased excitability via activation of disinhibitory circuits [88,89]. Reports in GAERS do indicate increased neuronal excitabilities in deep cortical layers associated with absence seizures [38,90]. Given the complexity of the cortical inhibitory microcircuits, a loss of cortical PV^+^-mediated FFI coupled with a further loss of phasic inhibition could cause increased excitability of principal excitatory neurons, which then leads to hyper-activation of disinhibitory motifs with subsequent downstream saturation of the extracellular space with GABA neurotransmitter. Any increase in extracellular ambient GABA would translate into increased tonic inhibition by activation of extra-synaptic GABA_A_Rs. Interestingly, we recently reported a significant increase in total GABA levels in the stargazer SoCx using HPLC, but a decrease in GABA expression within presynaptic terminals [91]. Moreover, we also found a significant increase in the GAD65 enzyme, but not GAD67 or GAT-1 (GABA Transporter-1) in the same region [92]. GAD65 is chiefly localized in the axon terminals [93], with increased GABA synthesis during intense synaptic activity mediated by this enzyme [94]. These findings substantiate the inference that there may be an extracellular increase in GABA in the epileptic primary SoCx. 

### 3.4. Conclusions

Mounting evidence suggests the importance of GABAergic inhibitory malfunction in the pathogenesis of absence seizures [1]. In this study, we primarily aimed to investigate changes in the ubiquitous GABA_A_R α1 subunit as a means of investigating GABA_A_R expression in the primary SoCx of the stargazer mouse model of absence epilepsy. GABA_A_R-mediated phasic and tonic inhibitory currents are integral to the physiological E/I balance of neuronal circuits and any change can alter the function, kinetics, and pharmacology of GABA_A_Rs [79]. GABA_A_R plasticity is a natural response of neuronal circuits perhaps as an attempt to balance hyperexcitability, but such changes can themselves contribute to further pathogenesis of the original disorder [95]. A loss of the GABA_A_R α1 subunit has been reported in the literature, both in human patients [96,97], as well as in genetic absence seizure animal models used in research. In the stargazer, loss of FFI coupled with the loss of synaptic GABA_A_R α1 could lead to enhanced excitability of target principal pyramidal neurons. EM assessment in this study, which analyzed GABA_A_R α1 subunit density within synapses (immunogold/μm length of synapse), revealed no change in the α1 subunit expression within the inhibitory synapses investigated. Conversely, semiquantitative WB analyses, which analyzed the subunit collectively at all synapses in the primary SoCx, revealed reduced α1 subunit expression. This could reflect a change in the number of inhibitory synapses or altered GABAergic connectivity from cortical GABAergic interneurons. The resulting enhanced cortical excitability could potentially alter the glutamate and GABA neurotransmitter dynamics that may exacerbate seizure generation. In the stargazer mouse model, genetic mutation has not only been associated with altered expression of GABA_A_Rs, but altered GABA neurotransmitter dynamics as well [34,78], which in turn can affect the development of neurons, synapses, and the expression of GABA_A_Rs themselves [98]. Further in-depth investigations into discerning the specific changes in GABAergic neurotransmission could be crucial for enabling development of targeted preventative and treatment strategies.

## 4. Materials and Methods

### 4.1. Animals

The Stargazer mice *(B6C3Fe a/a-Cacng2^stg^/J)* used in this study were derived from breeding stocks obtained from the Jackson Laboratory (Bar Harbor, ME, USA). Adult 8–12-week-old epileptic (E) stargazers (stg/stg) and their NE (heterozygous [Het, +/stg] and wild-type [WT, +/+)]) control littermates were raised at the University of Otago’s Animal Resource Unit. The mice had ad libitum access to food and were housed in well-ventilated cages with optimum environmental conditions (12 h light/dark cycle, temperature ~21 °C and ~50% humidity). Genotypes were confirmed from ear-notches taken post-sacrifice using common forward (TAC TTC ATC CGC CAT CCT TC), wild-type reverse (TGG CTT TCA CTG TCT GTT GC), and mutant reverse (GAG CAA GCA GGT TTC AGG C) primer sequences. All animal procedures were carried out according to the University of Otago Animal Ethics Committee approved protocols and guidelines (32/16). A total of 142 animals (77 NE control littermates and 65 epileptic stargazers) were used for all the experiments in this study. The ‘n’ numbers represent the sample numbers used within each experiment.

### 4.2. Immunofluorescence Confocal Microsocopy

Animals (3 control littermates and 3 stargazer mice) were perfuse-fixed with 4% PFA and brains extracted for confocal microscopy as previously described [28]. Following overnight post-fixation at 4 °C and cryoprotection in increasing concentrations of sucrose, the brains were coronally sectioned at 30 μm in a freezing cryostat. Brain slices were selected from the primary SoCx regions based on bregma points [99], from anterior (1.10 to 0.02), mid (0.02 to −0.82), and posterior (−0.82 to −1.70) regions. The sections were triple-labelled with GABA_A_R α1 (1:500; AGA-001, Alomone, Jerusalem, Israel), PV (1:2000; PV 235, Swant, Burgdorf, Switzerland), and VGlut2 (1:500; 135404, Synaptic Systems, Goettingen, Germany). Sections were labelled for GABA_A_R α1 to confirm its distribution in the stargazer primary SoCx against NE littermates and to confirm the presence of α1 subunit across the layers. The tissue was also probed for PV protein to assist in recognizing GABA_A_R α1 in PV^+^ neurons [21]. To help distinguish the cortical layers, the tissue was also labelled for VGlut2, which causes intense labelling of thalamocortical projections in layer IV [49,51,100], and has previously been used by us for the same purpose [21]. GABA_A_R α1 pre-adsorption and omission control sections were processed in parallel (Supplementary Figure S1). After development of immunofluorescence with Alexa Fluor 568 (1:1000; A-11031, Life Technologies, CA, USA), Alexa Fluor 488 (1:1000; A-11008, Life Technologies, CA, USA), and Alexa Fluor 633 (1:1000, A21105, Life Technologies, CA, USA), images were acquired using Nikon A1+ Inverted Confocal Laser Scanning Microscope. The following channel configuration was used: green channel 488 nm for GABA_A_R α1, red channel 543 nm laser for PV and blue channel 633 nm laser for VGlut2. Z-stacks of primary SoCx were taken from each section at different planes, with the aim of at acquiring between 6 and 9 images in each stack. Optimal planes were determined by the brightest fluorescence. Laser intensity, gain, and offset were determined using the white matter and maintained across all image acquisition. The numerical aperture was kept at 1.3, pinhole size was set to 1 AU, and z-axis step-size was chosen as 1 μm. Image format was acquired at 1024 × 1024 pixels for 10× magnification (Objective: 10× Plan), and 512 × 512 for 60× magnification (Objective: 60× PlanApo oil). Images were observed using ImageJ software (NIH, Bethesda, MD, USA). Channels were first split and then enhanced using ImageJ Z-project and flatten functions.

### 4.3. Western Blotting

Mice were decapitated after sacrifice through cervical dislocation, brains extracted and snap-frozen on dry ice. Brains were sectioned in a cryostat (Leica CM1950, Leica Microsystems, Wetzlar, Germany), maintained at −10 °C, and 300 μm thick coronal sections produced, which were thaw-mounted onto glass slides followed by microdissection of the primary SoCx region out of each section between bregma 1.09 to bregma −1.91. Tissue samples obtained were subsequently processed for Western blotting. Briefly, micro-dissected tissue samples were immediately placed into lysis buffer (0.5 M Tris-base, 100 mM EDTA, 3% SDS, pH6.8) supplemented with 1% phenylmethyl sulfonyl fluoride (PMSF) and 1% protease inhibitor (P8340, Sigma-Aldrich, St. Louis, MO, USA). Tissue samples were homogenized via sonication, heated for 2 min at 100 °C, and centrifuged at 13,000 RPM for 5 min in a chilled centrifuge (4 °C) to extract the supernatants, which were stored at −80 °C. The samples were quantified for protein content using detergent-compatible colorimetric assay (DC Protein Assay, 500, 0116, Bio-Rad, Hercules, CA, USA). The protein content in the samples were separated in an 8.5% resolving gel using SDS-PAGE, followed by transfer onto nitrocellulose membranes. Membranes were probed using GABA_A_R α1 (1:500; AGA-001, Alomone, Jerusalem, Israel), GABA_A_R α3 (1:500; AGA-003, Alomone, Jerusalem, Israel), GABA_A_R α4 (1:200; AGA-008, Alomone, Jerusalem, Israel), GABA_A_R α5 (1:1000; AB9678, Sigma-Aldrich, St. Louis, MO, USA), GABA_A_R β2 (1:1000; ab8340, Abcam, Cambridge, UK), GABA_A_R β3 (1:1000; ab4046, Abcam, Cambridge, UK), GABA_A_R γ2 (1:200; AGA-005, Alomone, Jerusalem, Israel), and GABA_A_R δ (1:200; AGA-014, Alomone, Jerusalem, Israel) with β-actin (1:1000; ab8226, Abcam, Cambridge UK) used for normalization. After enhancement of immunofluorescence with IRDye800CW (1:1000; 926-32221, LI-COR Biosciences, Lincoln, NE, USA) and IRDye680 (1:1000; 926-32210, LI-COR Biosciences, Lincoln, NE, USA) secondary antibodies, membranes were scanned using Odyssey Infrared Imager (Li-Cor Biosciences, Lincoln, NE, USA) and protein bands were analyzed for integrated intensity using Odyssey v3.1 software (Li-COR Biosciences, Lincoln, NE, USA). Representative full blots are provided in supplementary Figure S2 (GABA_A_R α1 and α3), Figure S3 (GABA_A_R β2, β3, and γ2), and Figure S4 (GABA_A_R α4, α5 and δ). GABA_A_R subunit intensity values were normalized against β-actin control bands, and ratios of the normalized intensities against mean normalized values of NE littermates within each litter was calculated before statistical testing and graphical representation of data.

### 4.4. Biochemical Fractionation

Primary SoCx microdissections were obtained from snap-frozen brains extracted from animals using the same process as described for Western blotting. Tissue samples were homogenized in ice-cold fractionation buffer (320 mM sucrose, 10 mM tris-base, 1 mM EDTA, pH 7.37) supplemented with PMSF and protease inhibitor using sterile plastic pestles and sonication. A multistep centrifugation technique was used to isolate subcellular fractions from homogenized samples as previously described [20]. Briefly, each tissue sample was centrifuged at 1000× *g* for 10 min to obtain supernatant (total lysate), followed by further centrifugation of the extracted supernatant at 10,000× *g* for 15 min to separate out the membrane component as pellet. The fresh supernatant was separated out as cytosol fraction, while the membrane pellet was re-suspended in homogenization buffer (50 mM Tris, 2 mM EDTA, 3% SDS, pH 6.8) containing triton X-100, followed by incubation on ice for 40 min, and centrifugation at 32,000× *g* for 30 min to isolate the synaptic component as pellet. This separation depends on the insolubility of the synaptic densities in triton X-100. The supernatant-containing extra-synaptic component was extracted while the pellet was re-suspended in the homogenization buffer. The supernatant (extra-synaptic fraction) was mixed overnight with acetone, followed by 15 min 3000× *g* centrifugation to isolate the extra-synaptic component as pellet which was re-suspended in the homogenization buffer. The subcellular fractions were processed and analyzed as described above in Section 4.3. PanC (1:1000; 4068P, Cell Signaling Technology, Danver, MA, USA) was added for normalization and analyses while PSD-95 (1:1000; 124011, Synaptic Systems, Goettingen, Germany) and β-actin were used as additional controls. Representative full blots are provided in Supplementary Figure S5 (GABA_A_R α1 and α3) and Figure S6 (GABA_A_R α4, α5 and δ). The purity of the subcellular fractions was assessed (Figure 6). The GABA_A_R subunit intensities were normalized against Pan-C followed by relative ratios of the normalized intensities against mean normalized values of NE littermates within each litter calculated before statistically comparing stargazers against control littermates.

### 4.5. ICC-EM

Seven pairs of control littermates and epileptic stargazers were selected for this technique. The experiment was conducted with the researcher blinded to the identity of the samples. Samples were prepared as described previously [19,20,28,91]. Briefly, animals were sacrificed by trans-cardiac perfusion with 4% PFA and 0.1% glutaraldehyde in 0.1 M phosphate buffer (PB). The extracted brains were post-fixed in 4% PFA/0.1% glutaraldehyde followed by obtaining 250 μm thick coronal sections. Next, 1 mm wide micro-punches were taken from full thickness primary SoCx. Ultrathin sections mounted on formvar-coated nickel grids were prepared after sequential freeze-slamming, ultra-low temperature dehydration, cryo-substitution, and embedding in lowicryl HM20 resin of the tissue samples. Grid-mounted tissue was etched with sodium ethanolate for 3 s followed by blocking in 10% NGS for 2 h and overnight incubation in a primary antibodies cocktail of GABA_A_R α1 (1:50; AGA-001, Alomone, Jerusalem, Israel) and PV (1:50; 235, Swant, Burgdorf, Switzerland) to conduct double-labelling. The primary antibodies were probed with 10 nm immunogold (1:20; British Biocell International, Cardiff, UK) and 20 nm immunogold (1:20; British Biocell International, Cardiff, UK) secondary antibodies for GABA_A_R α1 and PV, respectively. The tissue was stained with uranyl acetate and lead citrate before imaging using a transmission electron microscope (Phillips CM100, Phillips/FEI corporation, Eindhoven, Holland). Grids were imaged at lower magnification to ascertain the layout of the tissue. This was followed by sequential imaging of synapses at high magnification. Inhibitory synapses were identified by their symmetrically thin, dense membrane ultrastructure, as can be seen in Figure 8. Excitatory synapses with their asymmetrical postsynaptic densities were clearly distinguishable from their inhibitory counterparts. Unbiased sampling was conducted throughout the tissue specimens by imaging all GABA_A_R α1-positive inhibitory synapses with at least 40 imaged for each animal. Synapses were subsequently analyzed in conjunction with PV^+^ profiles. Using ImageJ, the length of each inhibitory synapse was measured while counting all 10 nm gold particles confined to within 30 nm of synaptic density. Density of GABA_A_R α1 was obtained by dividing the number of gold particles against the length of synapses in which they were found. 

### 4.6. Statistical Analyses

Data were statistically analyzed using unpaired non-parametric Mann–Whitney U test for comparison between the two independent groups of control littermates and epileptic stargazers in the same population. Independent variability between the two groups is the existence of genetic mutation-based phenotype in the stargazers and lack thereof in the control group. Data from stargazers were first normalized against the controls before transferring into GraphPad Prism (GraphPad Software Inc., San Diego, CA, USA) for statistical analysis with statistical significance defined as: *p* < 0.05 as *; *p* < 0.01 as **; *p* < 0.001 as ***. The data in this study have been presented as mean ± standard error of mean (SEM).

## Data Availability

Data are accessible upon reasonable request.

## Supplementary Materials

The supporting information can be downloaded at: https://www.mdpi.com/article/10.3390/ijms232415685/s1.
