# Supplementary material for: Altered GABAA Receptor Expression in the Primary Somatosensory Cortex of a Mouse Model of Genetic Absence Epilepsy
Source: Int J Mol Sci. 2022 Dec 10;23(24):15685. doi: 10.3390/ijms232415685 (PMC9778655; doi:10.3390/ijms232415685)
Supplement: Supplementary file 1 [file ijms-23-15685-s001.zip › ijms-2053485-supplementary.pdf]

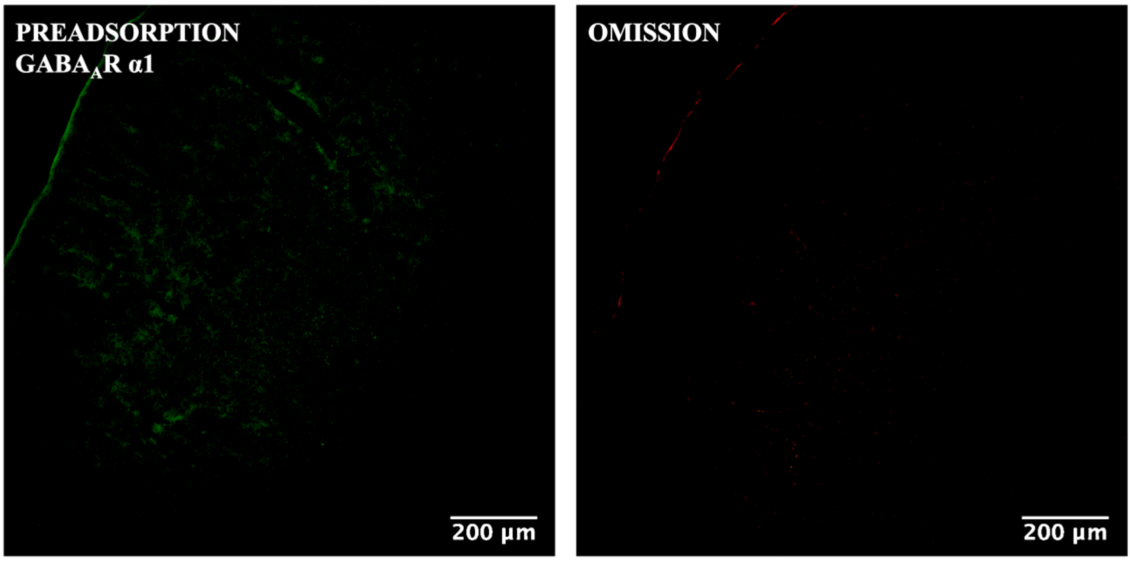

Figure S1. Representative control images for immunofluorescence confocal microscopy. Pre-adsorption control of tissue was conducted for GABAAR α1 primary antibody only. Omission controls show a virtual lack of fluorescence after subtraction of background intensities.

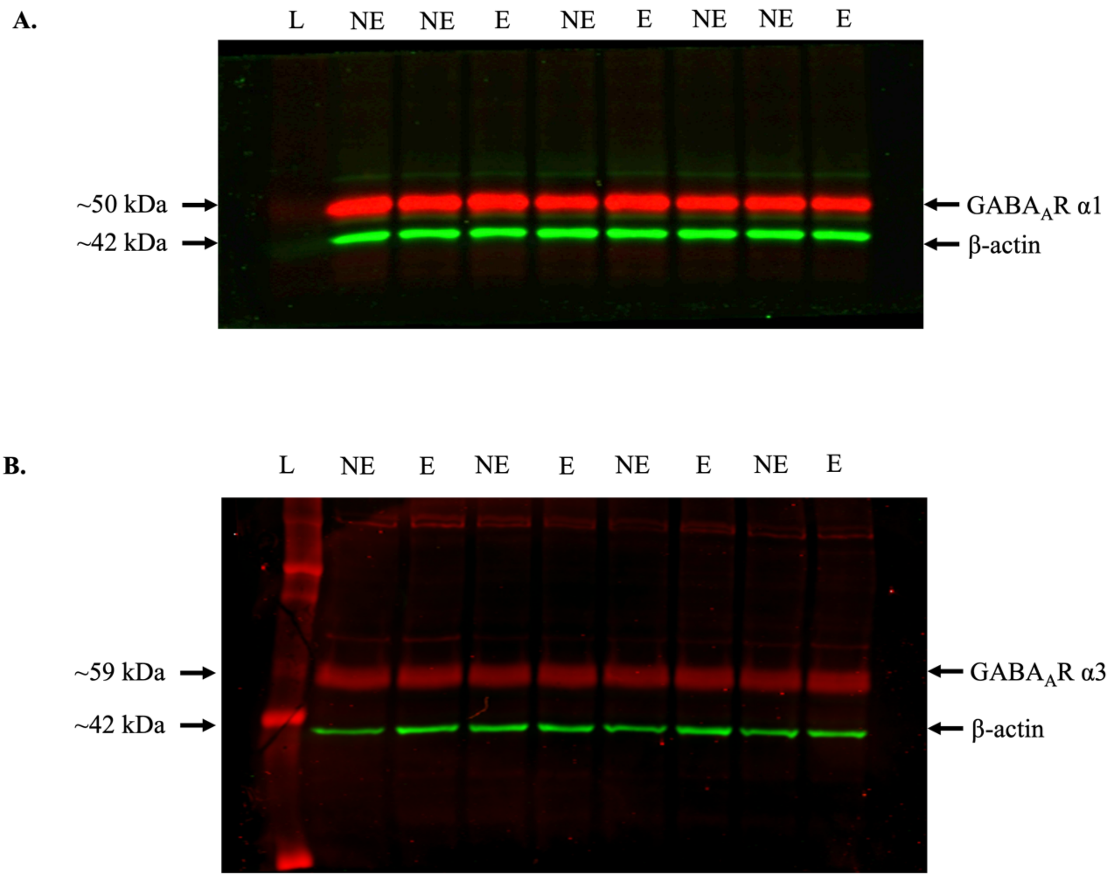

Figure S2. Representative whole western blots for global whole tissue assessment of primary somatosensory cortex for GABA$_A$R α1 (**A**) and α3 (**B**) in adult epileptic stargazers compared to their non-epileptic control littermates. (NE: Non-epileptic controls; E: epileptic stargazers)

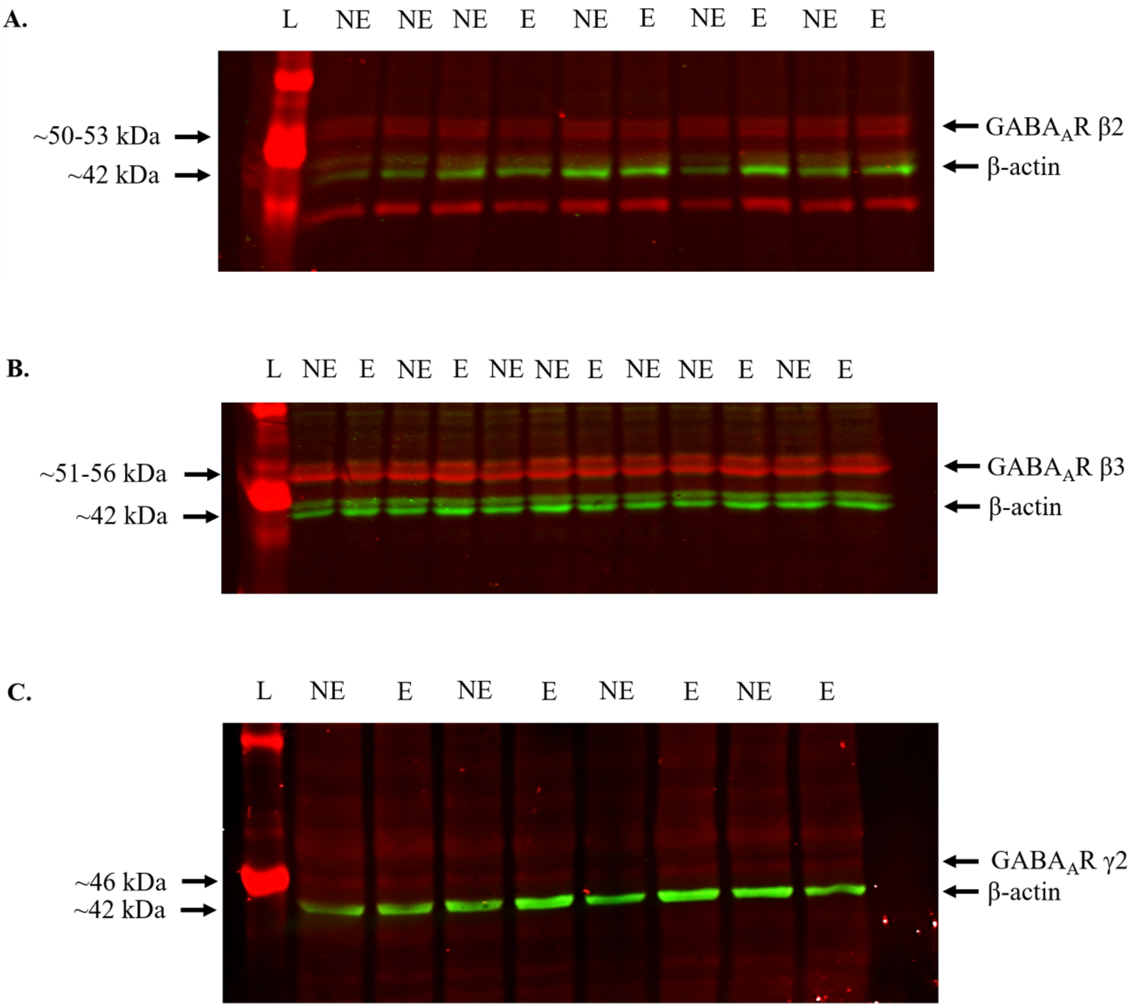

Figure S3. Representative whole western blots for global whole tissue assessment of primary somatosensory cortex for GABA$_A$R β2 (**A**), β3 (**B**) and γ2 (**C**) in adult epileptic stargazers compared to their non-epileptic control littermates. For GABA$_A$R γ2 the intensity of the band was quite low but was detectable at the manufacturer's recommended molecular weight upon converting the image to greyscale colour profile (NE: Non-epileptic controls; E: epileptic stargazers)

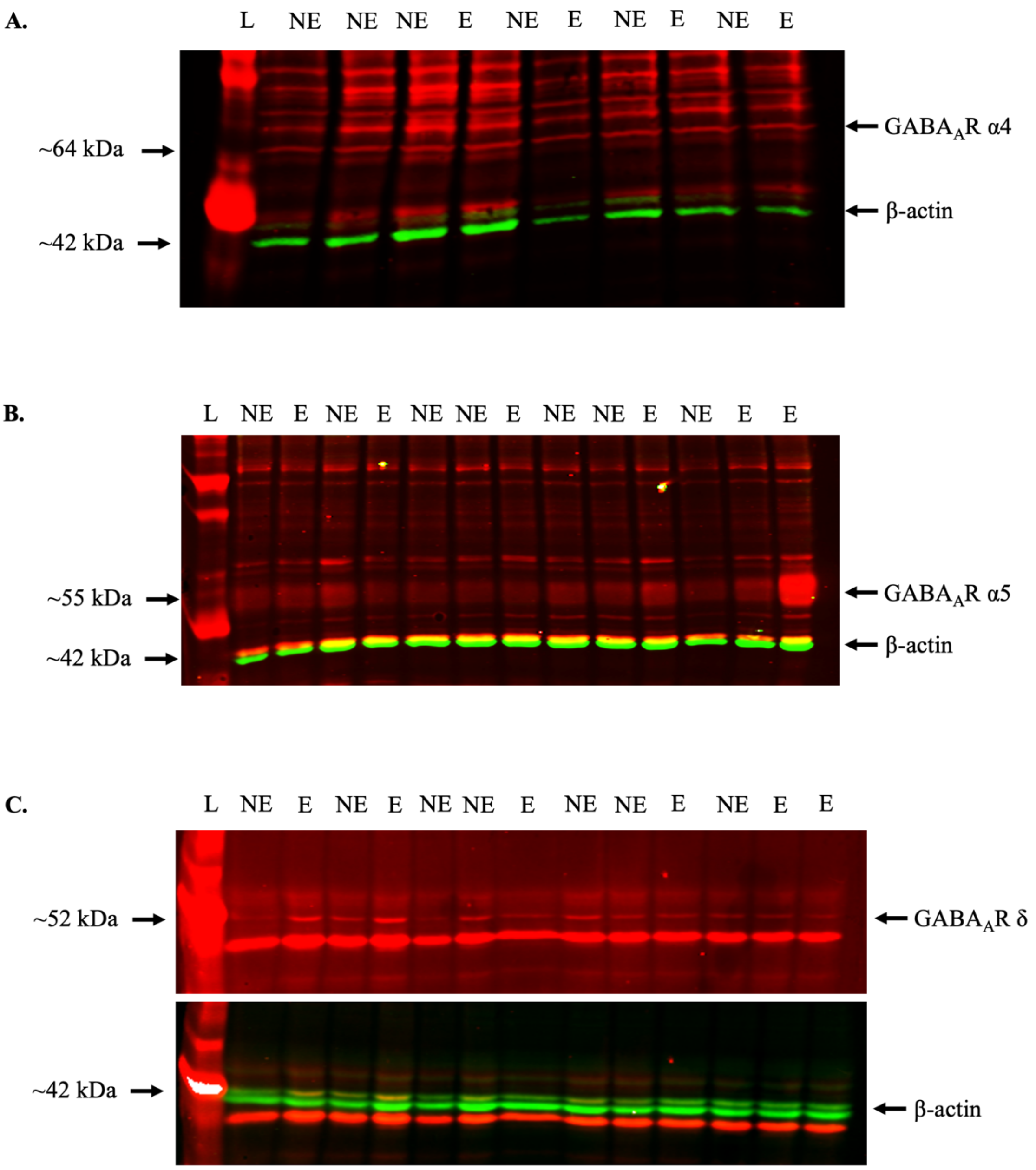

Figure S4. Representative whole western blots for global whole tissue assessment of primary somatosensory cortex for GABA$_A$R α4 (**A**), α5 (**B**) and δ (**C**) in adult epileptic stargazers compared to their non-epileptic control littermates. For GABA$_A$R δ during image scanning the red channel was imaged separately to visualize the band at the manufacturer's recommended molecular weight. (NE: Non-epileptic controls; E: epileptic stargazers)

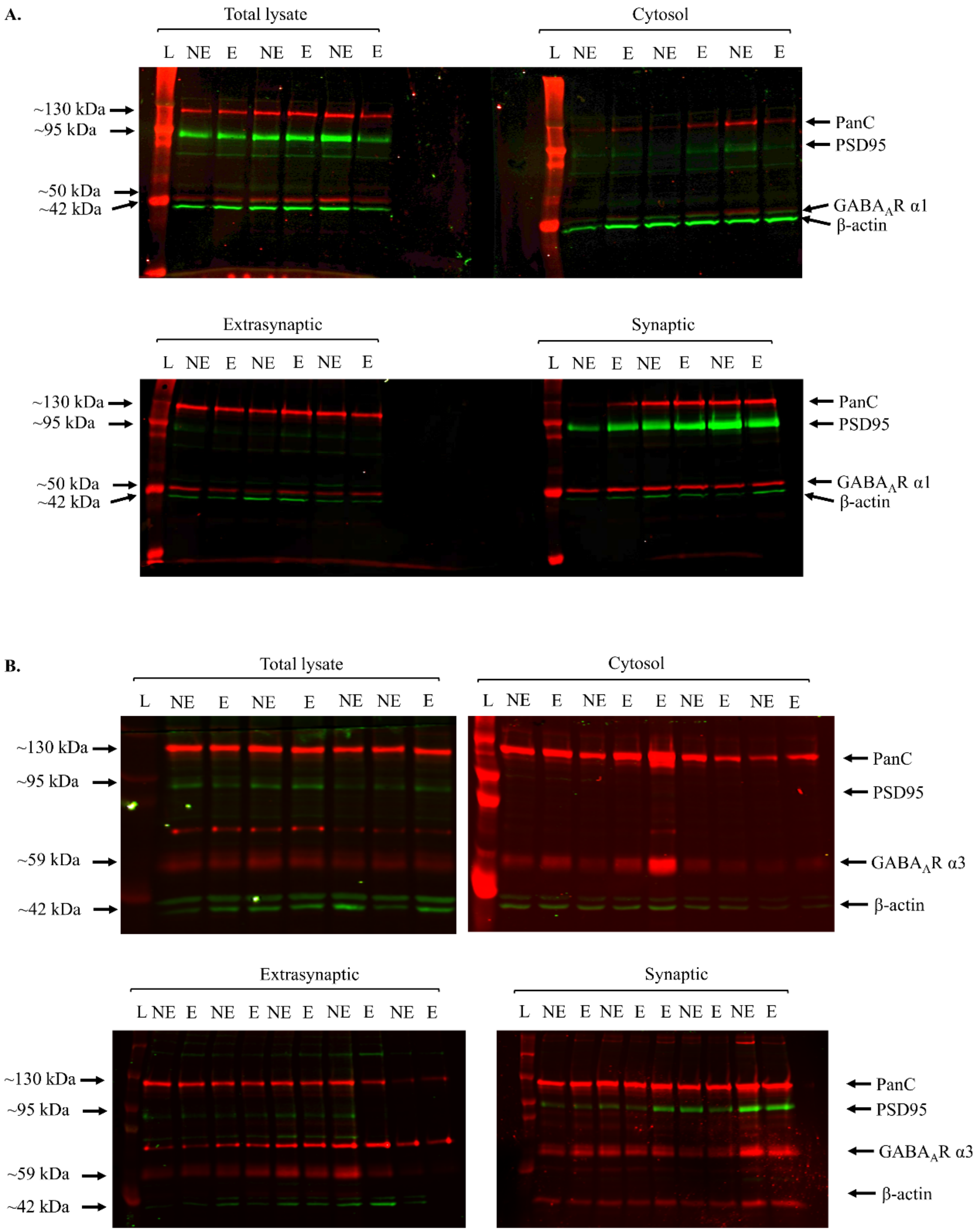

Figure S5. Representative western blots for subcellular fractions (total lysate, cytosol, extra-synaptic and synaptic) from primary somatosensory cortex of adult epileptic stargazers and non-epileptic control littermates. The subcellular fractions were probed for GABA$_A$R α1 (**A**) and α3 (**B**) with PanC for normalisation and analyses. PSD95 labelling was used to confirm the synaptic fraction. For analyses the bands were selected at the manufacturers recommended molecular weight for the antibodies used with the help of the protein ladder. (NE: Non-epileptic controls; E: epileptic stargazers)

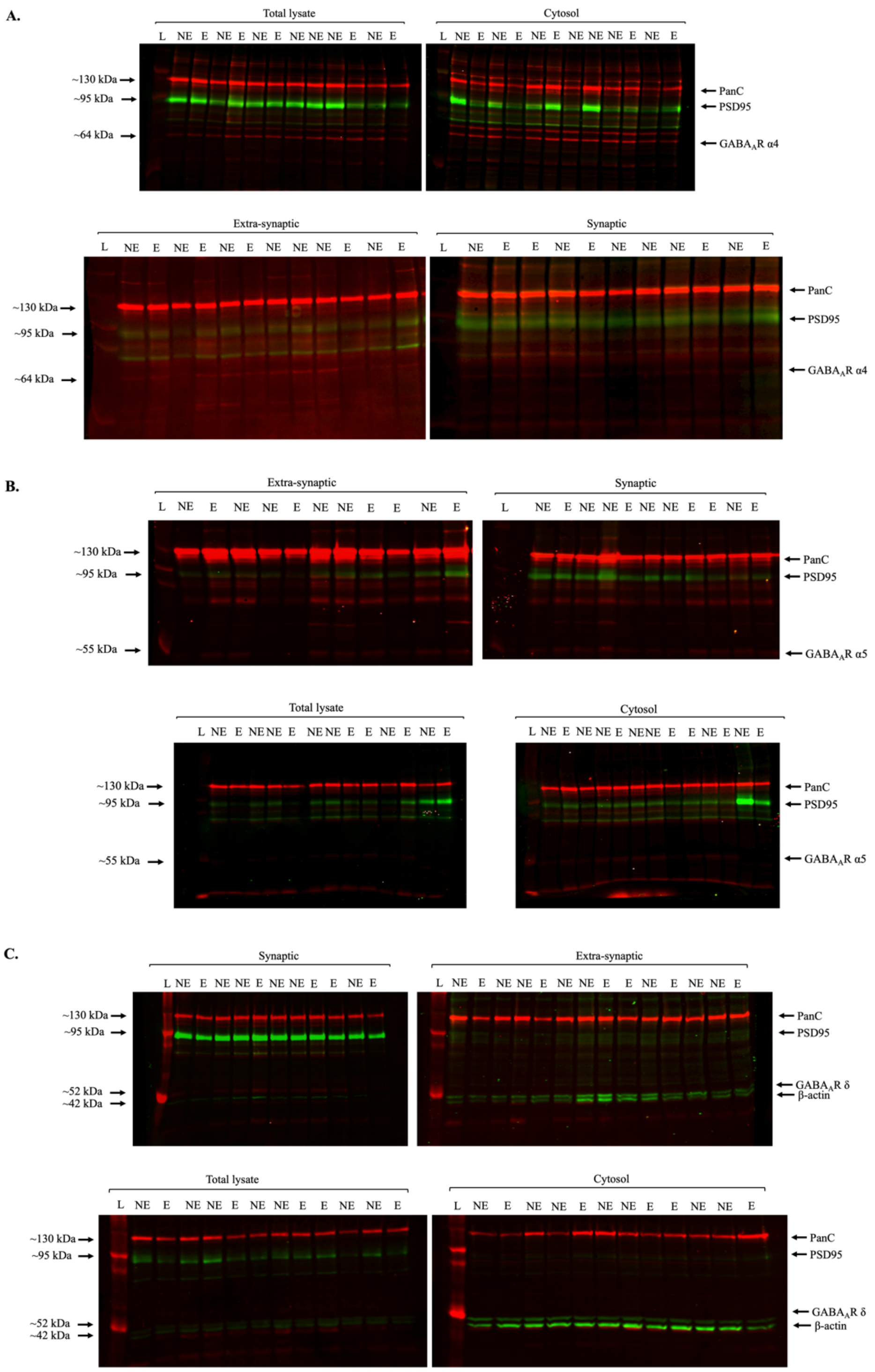

Figure S6. Representative western blots for subcellular fractions (total lysate, cytosol, extra-synaptic and synaptic) from primary somatosensory cortex of adult epileptic stargazers and non-epileptic control littermates. The subcellular fractions were probed for GABA$_A$R $\alpha4$ (**A**), $\alpha5$ (**B**) and δ (**C**) with PanC for normalisation and analyses. PSD95 labelling was used to confirm the synaptic fraction. For analyses the bands were selected at the manufacturers recommended molecular weight for the antibodies used with the help of the protein ladder. Low intensity GABA$_A$R subunit bands were visualised by isolating the manufacturer's recommended molecular weight section of the blot using odyssey infrared imager. (NE: Non-epileptic controls; E: epileptic stargazer
